# Physical properties and biological effects of mineral trioxide aggregate mixed with methylcellulose and calcium chloride

**DOI:** 10.1590/1678-7757-2017-0050

**Published:** 2017

**Authors:** Bin-Na Lee, Soo-Ji Chun, Hoon-Sang Chang, Yun-Chan Hwang, In-Nam Hwang, Won-Mann Oh

**Affiliations:** 1 Chonnam National University, School of Dentistry, Dental Science Research Institute, Department of Conservative Dentistry, Gwangju, Korea

**Keywords:** Flowability, Methylcellulose, MTA, Osteogenic differentiation

## Abstract

**Objectives:**

Methylcellulose (MC) is a chemical compound derived from cellulose. MTA mixed with MC reduces setting time and increases plasticity. This study assessed the influence of MC as an anti-washout ingredient and CaCl_2_ as a setting time accelerator on the physical and biological properties of MTA.

**Material and Methods:**

Test materials were divided into 3 groups; Group 1(control): distilled water; Group 2: 1% MC/CaCl_2_; Group 3: 2% MC/CaCl_2_. Compressive strength, pH, flowability and cell viability were tested. The gene expression of bone sialoprotein (BSP) was detected by RT-PCR and real­ time PCR. The expression of alkaline phosphatase (ALP) and mineralization behavior were evaluated using an ALP staining and an alizarin red staining.

**Results:**

Compressive strength, pH, and cell viability of MTA mixed with MC/CaCl_2_ were not significantly different compared to the control group. The flowability of MTA with MC/CaCI_2_ has decreased significantly when compared to the control (p<.05). The mRNA level of BSP has increased significantly in MTA with MC/CaCl_2_ compared to the control (p<.05). This study revealed higher expression of ALP and mineralization in cells exposed to MTA mixed with water and MTA mixed with MC/CaCl_2_ compared to the control (p<.05).

**Conclusions:**

MC decreased the flowability of MTA and did not interrupt the physical and biological effect of MTA. It suggests that these cements may be useful as a root-end filling material.

## Introduction

Since its introduction as a root-end filling material and as a root perforation repair in 1993, the use of mineral trioxide aggregate (MTA) has been expanded to many clinical applications including pulp capping, pulpotomy, and apical barrier for teeth with necrotic pulp and open apices^5^ ^,^ ^12^ ^,^ ^13^ ^,^ ^15^ ^,^ ^26^ ^,^ ^34^. This popularity originates from the superior biocompatibility and sealing ability of MTA to other root-end filling materials^1^ ^,^ ^24^ ^,^ ^34^. However, it has several disadvantages, including long setting time and poor handling characteristics because of its granular consistency^23^ ^,^ ^33^.

There are some studies aimed at improving the setting time and handling characteristics of MTA by using certain additives^8^ ^,^ ^16^ ^,^ ^23^. The addition of amorphous calcium lactate gluconate based liquid improves the setting time as well as clinical manageability^16^, however, it decreases the compressive strength of MTA^25^. In another study, the addition of propylene glycol improved flowability and increased the pH and calcium ion release during the initial post-mixing periods, despite increasing its setting time^11^.

Methylcellulose (MC) is a chemical compound derived from cellulose. It is used as an additive to improve the performance of Portland cement in wet environments, as a thickener and emulsifier in several food and cosmetics, and also as a constipation treatment. Cellulose, for example, is not digestible, neither toxic, nor allergenic. An admix of 1% MC and 2% calcium chloride (CaCl_2_) into MTA reduced setting time and improved its moldability similarly to a reinforced zinc oxide-eugenol cement with an approximately equal compressive strength^6^. Moreover, the addition of 10% CaCl_2_ to MTA did not alter its biologic properties regarding the formation of a mineralized barrier after pulpotomy^8^ and MTA mixed with calcium compounds showed a similar inflammatory response to MTA mixed with sterile water in an *in vivo* study^29^. However, there are few studies on the physical properties and biological effect of MC on MTA. In addition, interaction between root-end Ailing materials and periradicular tissues is very important at the start and development of healing. Thus, the aim of this study was to investigate the influence of MC and CaCl_2_ (MC/CaCl_2_) on the physical and biological properties of MTA.

## Material and methods

### Preparation of test materials

To prepare the additive solutions, CaCl_2_ (Sigma, St Louis, MO, USA) with 2% of sample weight was added to distilled water and mixed into a solution. This CaCl_2_ solution was placed on a hot plate whose temperature was raised to 80°C. MC (Sigma) was added to the warm solution to obtain the concentrations to be tested (1% or 2%) and was stirred until all the materials were mixed. Then, the solution was stored at 0°C for 20 minutes to make it thicker and stirred with a magnetic stirrer for 30 minutes to create a homogenous gel. Similar to the manufacturer's recommendations for MTA (ProRoot MTA; Dentsply Tulsa Dental, Tulsa, OK, USA), these solutions were used in a 3:1 powder-liquid ratio and assigned to the following test groups. Group 1(control): distilled water; Group 2: 1% MC/CaCl_2_; Group 3: 2% MC/CaCl_2_.

### Compressive strength

The compressive strengths of the materials under test were determined according to the method recommended by the ISO 9917-1^20^. To prepare the specimens, polyethylene molds with 4 mm inner diameter and 6 mm height were used. The specimens were removed from the molds and a search for any air-voids or chipped edges was conducted. All defective specimens were discarded. Six samples were selected to undergo material testing, and prepared for each material test at each time interval (n=6). The specimens were immersed in distilled water for 1 day, 3 d, and 7 d and maintained at 37°C. The compressive strengths were then measured using a universal testing machine (RB Model 302 ML, R&B Inc., Korea) at a crosshead speed of 1.0 mm per m. The maximum load needed to fracture each specimen was measured, and the compressive strength (C) was calculated in megapascals according to the formula:

C=4P/ΠD^2^

where P is the maximum force applied in newtons, and D is the mean diameter of the specimen in millimeters.

### pH

The pH was measured by using a pH meter (ORION STAR A211; Thermo Scientific, MA, USA) with an electrode for solid specimens (InLab Surface; Mettler Toledo, Schwerzenbach, Switzerland). Before the test, the apparatus was calibrated at the standard pH solutions of 4.0, 7.0 and 11.0. Six specimens for each group were prepared by loading the material under test into acrylic molds with 10.0 mm inner diameter and 5.0 mm height (n=6). The readings were taken at the end of the mixing and after 30 m, 1 h, 3 h, 24 h, 48 h and 72 h. Between each measurement, the electrode was washed with distilled water and blot dried.

### Flowability

The flowability of each group was determined as recommended by ISO 6876^19^. Immediately after mixing, 50 pL of each material was placed on a flat glass slab (40x40x5 mm, 20 g weight). The second glass slab was positioned on the material followed by the addition of 100 g of weight after 180 s from the start of mixing. The weight was removed after 10 m of mixing. The maximum and minimum diameters of the circle formed by the material were measured. The mean diameter is considered as a measurement of the flowability only if the difference between the maximum and minimum diameters is within 1 mm. Six tests were carried out for each material (n=6).

### Cell culture

Mouse periodontal ligament (mPDL) cells were used for this study and cultured in Dulbecco's modified Eagle's medium (DMEM; Invitrogen, Carlsbad, CA, USA), supplemented with 10% fetal bovine serum (FBS; Invitrogen) and 1% antibiotics (100 U/mL of penicillin and 100 µg/mL of streptomycin, Invitrogen) at 37°C in a humidified atmosphere containing 5% CO_2_. Cells used in these experiments were between passages 4 and 7.

### Material extracts

MTA samples were mixed with distilled water, 1% MC/CaCl_2_ and 2% MC/CaCl_2_ in a 3:1 powder-liquid ratio according to the manufacturers' instructions. The materials were placed into a cylindrical polyethylene tube (5 mm in diameter and 3 mm in height). To attain complete setting, the samples were kept for 6 h at 37°C and 95% relative humidity. After setting, discs were exposed to ultraviolet light for 1 h for sterilization and transferred into 24-well culture plates. Discs were incubated in 1.5 mL DMEM containing 2% or 10% FBS and antibiotics at 37°C in a humidified atmosphere with 5% CO_2_ for 24 h. The extracts were collected and then Altered using 0.20 pm Alters (Minisart, Sartorius Stedim Biotech, Goettingen, Germany).

### Cell viability assay

The mPDL cells were seeded into 96-well culture plates at a density of 1x10^4^ cells *per* well and incubated in a growth medium (DMEM containing 10% FBS and 1% antibiotics) for 24 h for adhesion. The medium was replaced with 100 pL material extracts of experimental groups and incubated for 24 h. The mPDL cells and the growth medium were used for control. To compare dose-response relationships, the material extracts were gradually diluted in the growth medium to obtain 5 concentrations (1, 1/2, 1/4, 1/10, and 1/50). Ten microliter of the WST reagent (EZCyTox; Daeil Lab Service Co., Seoul, Korea) was added to each well and incubated at 37°C for 3 h. Optical densities were measured at 420 nm using a multiwell spectrophotometer (VERSAmax Multiplate Reader; Molecular Devices, Sunnyvale, CA, USA). The relative cell viability was calculated for each test material as mean percentage of the control.

### Reverse-transcription PCR and quantitative real-time RCR

The mPDL cells were seeded in 6-well plates at a density of 2x10^5^ cells *per* well and incubated in a growth medium for 24 h. The growth medium was replaced with a medium containing 1/4 concentration of material extracts. The untreated cells were used for control. After 1, 3, and 5 d in culture, the total RNA was extracted using a TRIzol reagent (Invitrogen). The purity and quantity of total RNA were determined using a spectrophotometer (Nanodrop 100; Thermo Fisher Scientific, Waltham, MA, USA). Complementary DNA was synthesized using the Maxime RT PreMix Kit (iNtRON Biotechnology, Seongnam, Korea). Each reaction consisted of an initial denaturation at 95°C for 1 m followed by a three-step cycling: denaturation at 95°C for 30 s, annealing at 55°C for 30 s, and extension at 72°C for 30 s. After 25-30 cycles, the reactions underwent a final extension at 72°C for 5 m. The primer sequences were the following: bone sialoprotein (BSP), forward 5'-ACACTTACCGAGCTTATGAG-3' and reverse 5'-TTGCGCAGTTAGCAATAGCA-3'; and ß-actin, forward 5'-TGGATGGCTACGTACATGGCTGGG-3' and reverse 5'-TTCTTTGCAGCTCCTTCGTTGCCG-3'. Each reaction was analyzed with 1.5% agarose gel electrophoresis and visualized with ethidium bromide staining. Quantitative real-time PCR was performed by using the QuantiTect SYBR Green PCR kit (Qiagen, Valencia, CA, USA). The mean cycle threshold values from triplicate measurements were used to determine the relative level of expression of the target gene with normalization to the endogenous control ß-actin. The relative change in gene expression was analyzed by the ΔΔCT method^27^.

### Alkaline phosphatase (ALP) staining

The mPDL cells were seeded in 24-well plates at a density of 2x10^4^ cells *per* well with growth medium. After 24 h, the growth medium was changed to a medium containing 1/4 concentration of material extracts and cultured for 5 d. The cultured cells were washed with phosphate buffered saline (PBS) and fixed with 70% ethanol for 20 m at 4°C. After fixation, the cells were washed 3 times with deionized water and 300 µL ALP staining solution (1-Step NBT/BCIP Solution, Thermo Fisher Scientific Inc., Rockford, IL, USA) was applied *per* well under dark conditions for 15 m. The stains were extracted with 10% (w/v) ceptylpyridinium chloride (Sigma) in 10 mM sodium phosphate for 15 m. The ALP stain was quantified by recording the absorbance at 562 nm using a spectrophotometer (VERSAmax multiplate reader, Molecular Devices).

### Alizarin red staining

Alizarin red staining was used to assess mineralized deposit formation. The mPDL cells were cultured with growth medium containing 1/4 dilutions of material extracts for 14 d. The cells were rinsed with PBS, and fixed with 70% ethanol for 60 m at 4°C. The cells were stained with 0.5% alizarin red (pH=4.2) for 60 m at room temperature with gentle agitation. The cells were then rinsed with deionized water 5 times, rinsed with PBS for 15 m and air-dried. 10% (w/v) ceptylpyridinium chloride (Sigma) in 10 mM sodium phosphate was applied to the stained nodule and the absorbance of the supernatants was recorded at 540 nm using a spectrophotometer (VERSAmax multiplate reader, Molecular Devices) for quantitative assessment.

### Statistical analysis

Each experiment, containing triplicate independent samples, was repeated at least twice, and qualitatively identical results were obtained. One-way analysis of variance (ANOVA) was usd followed by Tukey post hoc test to assess any differences in flowability and ALP activity and alizarin red staining. For compressive strength, pH and cell viability, the two-way ANOVA and the Duncan tests were used. Data from real­ time PCR was analyzed with the Kruskal-Wallis and Mann-Whitney U tests. The results were considered statistically significant at p<.05.

## Results

### Compressive strength

The compressive strengths of MTA mixed with 1% and 2% MC/CaCl_2_ were not significantly different compared to MTA mixed with distilled water (Table 1). Also, there was no statistically significant difference between the time points.

**Table 1:** Means and standard deviations of the compressive strengtth of test materials at various time intervals

Group	1 day	3 days	7 days
MTA + DW	18.88±4.53	22.71±3.25	20.96±4.97
MTA + 1% MC	19.19±3.06	23.95±7.43	25.40±7.03
MTA + 2% MC	24.19±5.46	22.13±4.46	22.24±4.59

### pH

The mean pH of all samples is in Table 2. The pH values of MTA mixed with 1% and 2% MC/CaCl_2_ were not significantly different compared toMTA mixed with distilled water. Up to 3 h, all groups showed higher pH values compared to the other periods, with a significant difference of p<.05. After 3 h, despite decreasing, the pH values still remained high.

**Table 2:** Means and standard deviations of the pH of test materials at various time intervals

Group	Immediately	30m	1h	3h	24h	48h	72h
MTA + DW	12.83±0.02^Aa^	12.90±0.04^Aa^	12.74±0.27^Aa^	11.82±1.03^Aa^	10.93±0.94^Ab^	9.82±0.33^Ac^	9.61±0.29^Ac^
MTA + 1% MC	12.95±0.01^Aa^	12.99±0.14^Aa^	12.76±0.22^Aa^	12.21±1.05^Aa^	9.99±0.54^Ab^	9.53±0.14^Ab^	9.43±0.15^Ab^
MTA + 2% MC	12.90±0.06^Aa^	12.97±0.04^Aa^	12.96±0.08^Aa^	12.57±0.36^Aa^	10.02±0.43^Ab^	9.61±0.19^Ab^	9.49±0.08^Ab^

* A capital letter indicates no significant difference between test materials (p>.05)

* Different lowercase letters indicate significant difference between periods (p<.05)

### Flowability

**Figure 1:**
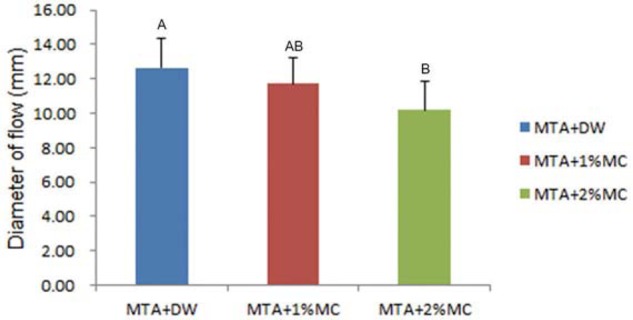
The flowability of test materials. The flowability of MTA mixed with 1% and 2% MC/CaCI_2_ decreased when compared to MTA mixed with distilled water. Different letters represent statistically significant differences between the tested materials (p<.05)

The results from the flowability test are shown in Figure 1. The mean diameter of group 1 (distilled water) was 12.65±1.72 mm, of group 2 (1% MC/CaCl_2_) was 11.70±1.51 mm, and of group 3 was 10.17±1.68 (2% MC/CaCl_2_). The flowability of groups 2 and 3 decreased when compared to group 1 and we observed a statistically significant difference between group 1 and group 3.

### Cell viability

**Figure 2:**
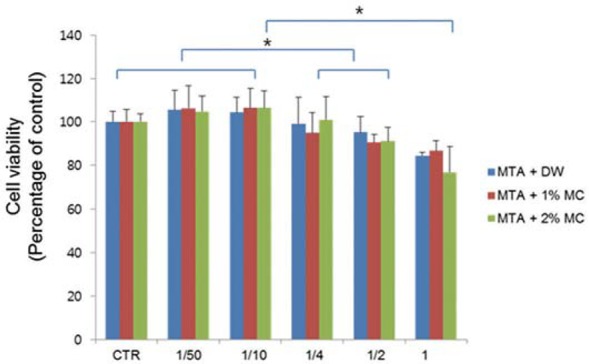
The cell viability of mPDL cells exposed to extracts of the test materials. The cell viability of MTA mixed with 1% and 2% MC/CaCl_2_ was not significantly different than that of MTA mixed with water. The cell viability of the control, and of 1/50 and 1/10 concentrations was significantly higher than that for 1/4,1/2 and 1 concentrations with the same material. And 1/4 and 1/2 concentrations had significantly higher cell viability than the 1 concentration (*p<.05)

The cell viability of MTA mixed with 1% and 2% MC/CaCl_2_ was not significantly different compared to MTA mixed with water (Figure 2). However, there were statistically significant differences in concentrations. The cell viability of the control, and of 1/50 and 1/10 concentrations were significantly higher compared to 1/4, 1/2 and 1 concentrations. And 1/4 and 1/2 concentrations had significantly higher cell viability comparedto the 1 concentration. There was no statistical difference between the tested materials. Based on this data, the 1/4 concentration was retained for the following experiments.

### RT-PCR and quantitative real-time RCR

**Figure 3:**
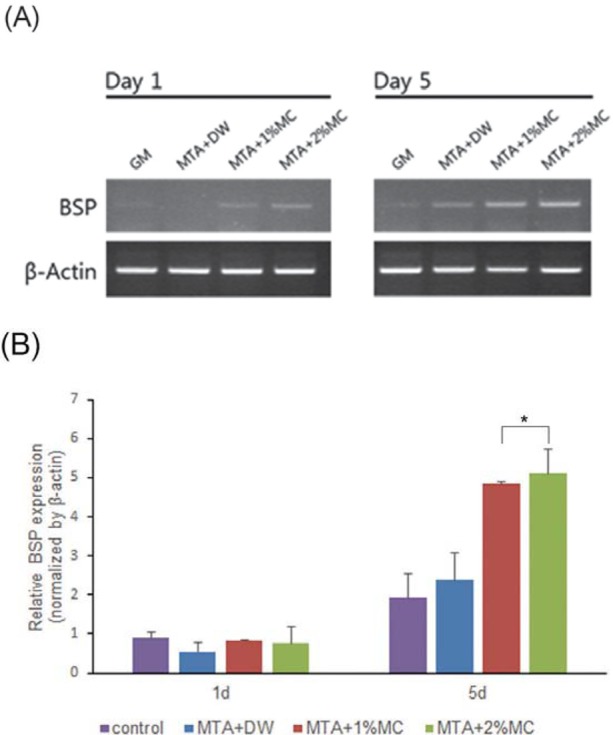
Expression profiles of bone sialoprotein (BSP) during osteogenic differentiation by test materials in mPDL cells assayed via RT-PCR and quantitative real-time PCR. (A) RT-PCR results. (B) The relative expression of BSP genes normalized against a housekeeping gene (ß-actin). The mRNA level of osteogenic gene increased in the MTA mixed with 1% and 2% MC/CaCI_2_. *p<.05, compared to the control

To investigate the effect of MTA mixed with MC/CaCl_2_ in osteogenic differentiation of mPDL cells, we evaluated the expression of BSP and the osteoblast-specific gene. As shown in Figure 3A, the expression of BSP mRNA increased in all tested groups compared to the control (treated with medium only) after 5 d. According to the results of real-time PCR, the mRNA level of BSP increased significantly in the MTA mixed with 1% and 2% MC/CaCl_2_ group compared to the control at day 5 (p<.05) (Figure 3B).

### Mineralization effect

**Figure 4:**
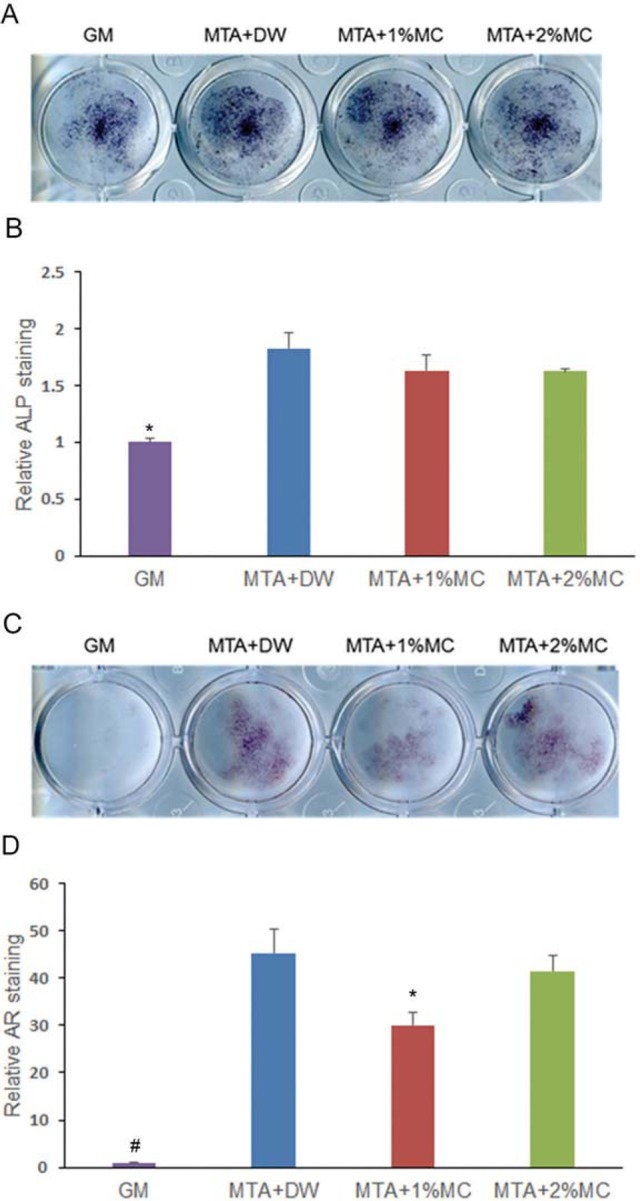
Alkaline phosphatase (ALP) staining and alizarin red staining in mPDL cells exposed to extracts of the tested materials. (A) Phenotype expression of ALP during osteogenic differentiation by the tested materials in mPDL cells at 5 d. (B) Quantified data. (C) Matrix mineralization of mPDL cells observed by staining calcium deposits in the extracellular matrix after 14 d. (D) Quantified data. We observed a higher ALP expression and mineralization in cells exposed to tested materials comparatively with the control. *p<.05, compared to the control

We investigated the mineralization effect of MTA mixed with MC/CaCl_2_ with ALP staining and alizarin red S staining. According to the results of ALP staining (Figure 4A and B), MTA mixed with distilled water and 1% and 2% MC/CaCl_2_ groups showed significant increase in ALP activity at 5 d compared to the control (p<.05). Alizarin red staining, used to detect mineralized nodule formation, showed all experimental groups increased mineralization significantly (p<.05) (Figure 4C and D).

## Discussion

MTA consists of fine hydrophilic particles of calcium silicates^21^. Despite MTA having many favorable properties, it has some disadvantages. It is difficult to mix MTA to an ideal consistency, to deliver it to the operation site, and to condense it densely. Several research has focused on these limitations^10^ ^,^ ^17^ ^,^ ^23^ ^,^ ^25^ ^,^ ^35^. Therefore, the objective of this study was to evaluate the physical and biological effects of MC that, when added to MTA, could improve its handling characteristics.

The compressive strengths of MTA mixed with 1% and 2% MC/CaCl_2_ were not significantly different compared to MTA mixed with distilled water. These values are similar to those reported by a previous study^23^. Compressive strength is an important factor to consider when placing a filling material in a cavity that bears pressure. Because MC does not influence the compressive strength of MTA, it can be used with MTA for perforation repair as well as root-end fillings. There were some studies on antimicrobial activity of MTA. They insisted that alkaline pH played an important role for this property^2^ ^,^ ^4^ ^,^ ^14^ ^,^ ^30^. In this study, the pH values of MTA mixed with 1% and 2% MC/CaCl_2_ were not significantly different compared to MTA mixed with distilled water. This pH stability at alkaline conditions may not affect the antimicrobial properties of the modified MTA system. MC consists of nonionic watersoluble cellulose ether and increases viscosity and dispersion resistance^3^. MC decreased the flowability of MTA significantly in this study. We first thought that MC improved the handling characteristics of MTA.

Considering it contains various stem cells^31^, we used the mPDL cell for several biological experiments such as cell viability, RT-PCR and mineralization assay of root-end Ailing materials in this study. The cell viability of MTA mixed with 1% and 2% MC/CaCl_2_ was not significantly different compared to MTA mixed with water. However, the cell viability of experimental groups in higher concentrations (1/4, 1/2, and 1 concentration) was significantly lower than the groups in lower concentrations (control, 1/50, and 1/10 concentration). Kang, et al.^22^ (2013) reported that MTA mixed with calcium chloride showed lower biocompatibility than MTA mixed with water. Therefore, calcium chloride added with MC might contribute to the lower cell viability of experimental groups in high concentrations. The differentiation of progenitor cells into osteoblast-like cells is critical in the healing process, and promoting differentiation is required when considering a biomaterial as a root-end filling material. BSP is the major phosphorylated protein of mammalian bone and its use has been suggested at the startof mineralization^7^ ^,^ ^18^. Consideringit is believed that this protein has a specific role in mediating the initial stage of mineralization^9^, increased BSP expression suggests the differentiation of several cells into osteoblasts. In this study, the expression of BSP mRNA increased in all tested groups compared to the medium-only treated group in RT-PCR. These results suggest that MTA stimulates the osteogenic differentiation of mPDL cells and MC did not interrupt the biological effect of MTA. Considering ALP and alizarin red staining data, it seems that MTA and MTA mixed with MC/CaCl_2_ stimulate the expression of ALP and the formation of calcium nodules in mPDL cells. These results also suggest that MTA stimulates the mineralization effect^28^ ^,^ ^32^ of mPDL cells and MC did not interrupt the mineralization effect of MTA.

These findings support the hypothesis that MC decreased the flowability of MTA and did not interrupt the physical and biological effects of MTA. However, there is need for further studies to clarify the detailed mechanism of how MTA and MTA mixed with MC/CaCl_2_ induce osteogenic differentiation of mPDL cells.

## Conclusion

MC decreased the flowability of MTA and did not interrupt its physical and biological effects, whichsuggests that these cements can be useful as root-end filing materials.
